# Unusual presentation of Stauffer syndrome in post Whipple patient: case report

**DOI:** 10.1016/j.eucr.2025.103065

**Published:** 2025-05-15

**Authors:** P.S. Harsha, Jinesh Jayadevan, C.H. Haris, Rajiv Thomas, H. Balabhaskar, Ramkumar Aiyappan, Sharoo Shaneej M

**Affiliations:** Department of Urology, Government Medical College, Trivandrum, India

**Keywords:** Stauffers syndrome, Renal cancer, Paraneoplastic syndrome

## Abstract

Stauffer syndrome is a rare paraneoplastic syndrome associated with Renal Cell Cancer (RCC), seen in 3 %–20 % of cases characterized by reversible nonmetastatic anicteric, hepatic dysfunction in the absence of direct hepatobiliary obstruction. We report a case of 78-yr old male, post Whipple surgery who presented with progressive and isolated elevation of Aminotransferases. Imaging confirmed the presence of a renal mass without hepatic metastasis. The patient underwent partial nephrectomy. The patient's clinical course, management, and subsequent resolution of hepatic dysfunction after resection underscore the importance of considering Stauffer syndrome in patients with unexplained liver test abnormalities.

## 1 Introduction

Stauffer syndrome is an uncommon liver-related paraneoplastic condition commonly associated with RCC.^1^ Similar hepatic abnormalities have also been described in connection with other tumors such as prostate cancer, bronchial adenocarcinoma, leiomyosarcoma, angiosarcoma, and malignant histiocytoma.^2^ It was first described in 1961 by Herbert Stauffer.^2^ Stauffer syndrome is also known as Block‐Stauffer‐Rothmand's Syndrome or Thomson‐Rothmand's Syndrome reflecting its evolving clinical descriptions since its initial publication by Stauffer in 1961. Almost all patients with Stauffer syndrome have an elevated serum alkaline phosphatase level, 67 % have elevated prothrombin time or hypoalbuminemia, and 20 %–30 % have elevated serum bilirubin or transaminase levels. Instead of intrinsic liver disease or tumor infiltration of the liver, this dysfunction is brought on by a paraneoplastic syndrome that, via a few hypothesised processes, results in aberrant bile flow.

## 2 Case report

A 79-year-old male with a past history of Whipple surgery for Duodenal Neuro Endocrine Tumour (NET)in 2015 presented to our OPD 9 years later with an incidental detection of Right Renal mass. Patient noticed a slight increase in his Liver Function Test in his routine blood examinations performed during his regular follow up visits in Gastro department. His Liver Function Test was Total Bil/Direct Bil- 0.9/0.3, Alkaline Phosphatase (ALP)- 127 U/l, Aspartate Aminotransferase (SGOT)- 89 U/l, Alanine Aminotransferase (SGPT)-115 U/l, ESR- 11, PT/INR- 18.1/1.13 sec. The patient had no history of fever, abdominal pain, vomiting and no history of addictions. On examination patient was a febrile, non-icteric, per abdomen was soft, non tender with no palpable mass.

**Fig. 1:**
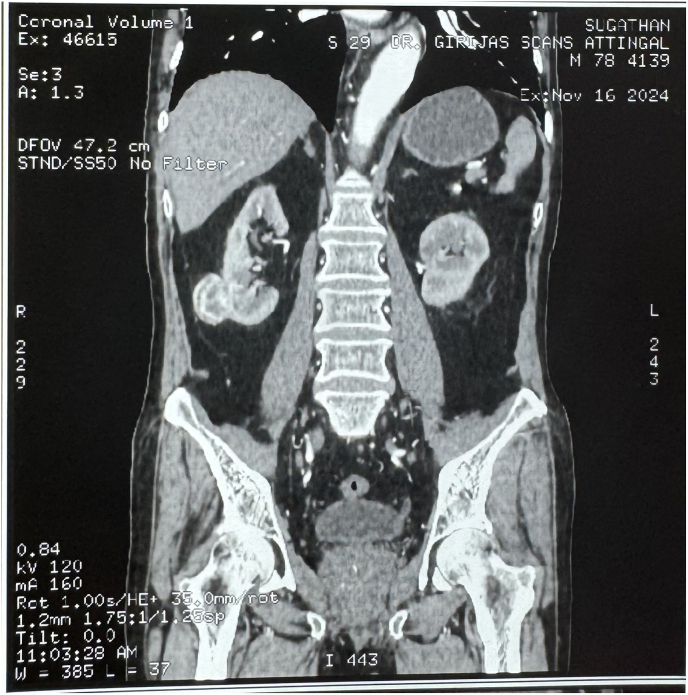
Computed tomography showing a well-defined hypo-to iso-dense exophytic lesion measuring 29 × 30 × 35 mm arising from the posterolateral aspect of the lower pole of the right kidney.

**Fig. 2:**
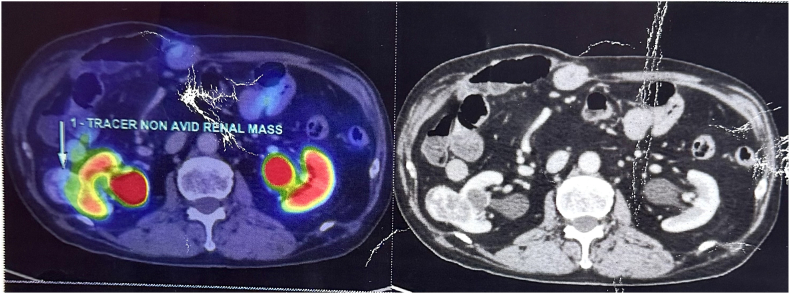
PET scan imaging showing a tracer non-concentrating, heterogeneously enhancing exophytic mass lesion arising from the interpolar region of the right kidney.

**Fig. 3:**
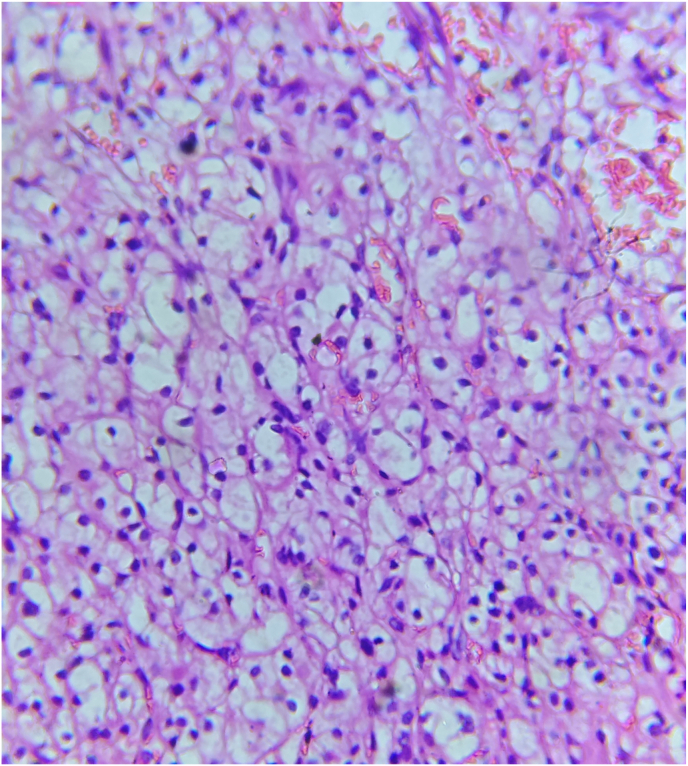
Histopathological image showing Clear cell RCC characterized by tumor cells with clear cytoplasm and distinct cell borders.

Patient was admitted for further evaluation and treatment. His SGOT and SGPT levels were found to be increasing progressively. From values of 89/115 U/l, it further changed to 104/141 U/l,153/262 U/l, 158/293 U/l,186/307 U/l, 370/403 U/l on serial follow-up. Blood test showed Gamma Glutamyl Transferase (GGT)- 23(0–55U/L), Alpha Feto Protein −1.9, Ceruloplasmin-25mg/dL (15–60), IgM HAV and IgM HEV negative, Anti HBc IgG – Negative. CECT abdomen (Fig. 1) and 68 Ga DOTATATE PET CT (Fig. 2) was taken. CECT abdomen showed a well-defined hypo to iso dense lesion of 29 ∗ 30 ∗35 mm arising exophytically from post-lateral aspect of Right kidney with moderate nodular heterogeneous post contrast enhancement, with no evidence of Hepatomegaly or NET recurrence. Patient was planned for Right Partial Nephrectomy. Surgery was performed without complications. Post operative period was uneventful. His SGOT/SGPT level reduced to 78/164 U/L on POD1. The patient was monitored closely in the postoperative period, with serial liver function tests showing a marked improvement. On Post Operative Day 5 (POD5) upon discharge, total bilirubin was 0.9 mg/dL, alkaline phosphatase 76 U/L, AST 68 U/L, ALT 32 U/L. Histopathology of the specimen showed Clear Cell renal cell carcinoma T1aN0M0(Stage 1) Grade 2 ISUP (Fig. 3), tumour size 3 × 2.5 × 2 cm with margins clear of neoplasm substantiating the diagnosis. At 3 weeks follow up, Liver function tests have become normalised.

## 3 Discussion

A rare paraneoplastic condition ^3^ known as Stauffer syndrome is characterized by involvement of the hepatic biliary system in the presence of visceral cancer, most frequently renal cell carcinoma. Stauffer syndrome illustrates a unique interplay between malignancy and systemic inflammatory responses. Its recognition is paramount, as it may be the first, and sometimes only, indicator of an underlying neoplasm. Several studies shows that Stauffer syndrome is associated with worse renal outcomes including high mortality, poor prognosis, and advanced renal cell carcinoma.^4^

Three pathophysiological mechanisms have been proposed. Primary tumor interleukin-6 over-expression is the most commonly accepted theory.^5^ In Stauffer syndrome, IL-6's pro-inflammatory action is thought to be the cause of cholestasis and hepatic dysfunction. IL-6 increases C-reactive protein and haptoglobin levels and also inhibits hepatobiliary transporter gene expression. Another theory that has been put forth to explain Stauffers syndrome is the colony-stimulating factor.^6^ An autoimmune aetiology was suggested as the third and least supported theory for these patients.^7^ There are two varieties of Stauffer's syndrome: the classical variant and the jaundice variant. The classic variant is characterized by cholestatic abnormalities in liver function tests, including α-2-globulin, γ-glutamyl transferase, alkaline phosphatase, hypoalbuminemia, thrombocytosis, and prothrombin time prolongation and elevated Erythrocyte Sedimentation Rate.^8^ Although the classical form is anicteric, clinicians should be aware of the potential for a jaundiced variant also. Crossovers can complicate the diagnosis of such a rare and frequently missed syndrome, which can be the only diagnostic clue for the detection of a hidden malignancy. A baseline liver function test, with special emphasis to bilirubin, ALP and GGT, should be the initial step toward diagnosis.^2^ To rule out any other underlying issue causing acute liver injury, a thorough liver function panel should be conducted. The GI team should also be consulted. Imaging should be used to confirm hepatosplenomegaly and rule out other causes, such as metastatic disease.^9^ A contrast-enhanced CT scan of the abdomen and pelvis can delineate renal mass, and evaluate for any associated hepatic, pancreatic, or metastatic malignancies. Disease management primarily consists of resection of the occult renal tumor, which is the source of the paraneoplastic syndrome, via nephrectomy after preoperative symptomatic care, such as ursodeoxycholic acid, cholestyramine, and avoidance of hepatotoxic medications. When faced with unexplained liver abnormalities, clinicians should be on the lookout for signs of renal cell carcinoma because it can manifest as a wide range of non-renal symptoms. In our patient, the progressive yet reversible derangements in liver function tests were the only clinical clues leading to further evaluation and eventual identification of an occult renal mass. Post-surgical improvement of liver enzymes following partial nephrectomy reinforces to a paraneoplastic mechanism underlying the hepatic dysfunction, as the removal of the primary tumor appears to mitigate the cytokine-driven process.

## 4 Conclusion

Stauffer Syndrome although rare, can be the only diagnostic symptom for early detection of Renal Cancer. Recent studies highlight its role as an indicator of recurrence by regular monitoring of liver function tests post nephrectomy. The prompt identification and surgical management of the renal tumor not only addressed the primary pathology but also resulted in the resolution of hepatic abnormalities. As such, clinicians should maintain a high index of suspicion for paraneoplastic syndromes in patients with unexplained liver function test elevations, as timely intervention can lead to significant clinical improvement and altered patient outcomes.

## CRediT authorship contribution statement

**P.S. Harsha:** Writing – review & editing, Writing – original draft, Supervision, Methodology, Investigation, Formal analysis, Conceptualization. **Jinesh Jayadevan:** Conceptualization. **C.H. Haris:** Writing – review & editing, Writing – original draft, Supervision, Conceptualization. **Rajiv Thomas:** Writing – review & editing, Project administration, Methodology. **H. Balabhaskar:** Writing – original draft. **Ramkumar Aiyappan:** Conceptualization. **Sharoo Shaneej M:** Writing – review & editing, Investigation.

## Consent

Informed and written consent was duly obtained for the publication of this case report and supplementary images.

## Disclosures

The authors have no conflict(s) of interest to disclose.

## Funding

This research did not receive any specific grant from funding agencies in the public, commercial, or not for profit sectors.
